# Concrete Obtained with the Viterbo O’Reilly Method for Aggregate Gradation: A Potential Model for Sustainable Design and Reducing Development Costs

**DOI:** 10.3390/ma18153558

**Published:** 2025-07-29

**Authors:** Edinson Murillo Mosquera, Sergio Cifuentes, Juan Carlos Obando, Sergio Neves Monteiro, Henry A. Colorado

**Affiliations:** 1 CCComposites Laboratory, Engineering School, Universidad de Antioquia UdeA, Calle 70 No. 52-21, Medellin 050010, Colombia; emurillom@unal.edu.co (E.M.M.); jcarlos.obando@udea.edu.co (J.C.O.); 2 Conasfaltos S.A., Medellín 051051, Colombia; scifuentesmosquera@gmail.com; 3 Military Institute of Engineering—IME, Praça General Tibúrcio 80, Urca, Rio de Janeiro 22290-270, RJ, Brazil; snevesmonteiro@gmail.com; 4 Facultad de Ingenieria, Universidad de Antioquia UdeA, Bloque 20, Calle 67 No. 53-108, Medellín 050010, Colombia

**Keywords:** aggregate gradation, concrete, Viterbo O’Reilly method, CO_2_ footprint

## Abstract

The following investigation presents concrete cement obtained with the Viterbo O’Reilly Diaz method, introduced to quantify the concrete mixture by using an aggregate gradation method. This research uses this procedure to decrease the amount of cement in the mix, thus reducing the CO_2_ footprint and production costs, which directly impact the environmental and economical sustainability of the material. The formulations used structural and general use Portland cements. As aggregates, fine sand and 3/4” gravel were included. Several characterization techniques were used, including granulometry testing for the aggregates, compression strength testing for the concrete samples, and granulometry testing for the raw materials. Compressive tests were conducted on samples after 28 days of curing, while scanning electron microscopy (SEM) with energy-dispersive spectroscopy (EDS) was used to understand the microstructure. The results revealed the optimal amounts of water, cement, and aggregates. Combinations of fine and coarse aggregates were determined as well. The main novelty in this manuscript is the use of the Viterbo O’Reilly mix design method to innovatively enhance concrete mixes by analyzing material properties and behavior in detail, an unexplored method in the literature. This research considers not only strength but also durability and workability, using mathematical tools for data analysis. This data-driven approach ensures effective aggregate gradation towards sustainability when compared to other traditional methods.

## 1. Introduction

Concrete is the most used material by weight worldwide, with about 14.0 billion m^3^ being used annually, corresponding to 4.2 billion tons and USD 440 billion, according to data from 2020 [1]. These numbers are quite important in all respects, considering that this industry has a significantly positive economic impact on human life but also a negative one when considering CO_2_ emissions [2], water consumption [3], and other environmental concerns. It is reported elsewhere that the concrete industry contributes to about 5–7% of the total CO_2_ generated by humankind, while nearly 900 kg of CO_2_ is released into the atmosphere as a byproduct of each ton of manufactured cement [4]. This has forced the construction sector to rapidly try to reduce this negative impact on the environment, using strategies as diverse as carbon capture and storage [5], supplementary cementing materials [6], nanomaterials [7], aggregate optimization [8], the use of construction and demolition waste and other residual materials [9,10,11], and lower clinker fabrication temperatures [12]. Alternatives such as alkaline-activated cements [13,14,15] and phosphate cements [16,17,18] have been proposed, but their applicability remains limited due to issues such as the limited availability of raw materials and high production costs, which are areas that represent some of the most significant advantages of Portland cement production.

Concrete is a complex multi-phase material obtained from the combination of water, cement powder, aggregates, and admixtures, and, therefore, the proportions of these components also impact the sustainability of this material. Concrete properties are highly influenced by the type and quality of aggregates, such as their mechanical strength or stability in environmental conditions.

Aggregate gradation is therefore a topic of great interest in the concrete industry, and there are several methodologies involving modern criteria and methods, such as multi-criteria decision-making strategies [8], to evaluate environmental, mechanical, and economic aspects, including the CO_2_ footprint, while maintaining material performance. Other approaches include the aggregate bearing capacity [19] based on macroscopic and mesoscopic aggregate contact forces studied through experiments and simulations; density-based topology optimization [20] with maximum size constraints; and an approach based on aggregate characteristics [21], such as size and shape, showing that the maximum stress and elastic modulus occur when the coarse aggregate content is 45%, the maximum size is 16  mm for coarse aggregate, and 75% of the coarse aggregates are rounded.

Several aggregate gradation methods have been extensively used by industry, with methods such as those by Fuller [22], Shilstone [23], Viterbo O’Reilly [24], and ACI [25] being among the most prominent. Fuller gradation [22] is based on maximum packing density theory, developed in 1907 and widely used elsewhere. The Shilstone gradation method [23] evaluates the impact of aggregates on concrete strength by categorizing them into coarse, intermediate, and fine fractions. ACI gradation [25] is a globally recognized method requiring aggregates to follow a defined granulometry curve. Viterbo O’Reilly gradation [24] focuses on minimizing void content through trial and error to identify the optimal mix of gravel and sand for maximum density. Other methods include the use of a gyratory compactor on aggregates [26], allowing for the development of a new laboratory design procedure for dense high-modulus asphalt concretes. Aggregate gradation driven by sustainability concepts is also gaining importance due to issues with pollution, water, and energy resources, with studies focusing on aggregate blends and packing for compressive strength and aggregate gradation enhancement [27].

Professor Viterbo O’Reilly [24] conducted significant technical research in the field of cement and concrete, focusing on improving material efficiency for concrete production. Confronted with the urgent need to reduce production costs in an economically constrained country like Cuba, Dr. Eng. O’Reilly developed and implemented a mix proportioning method. This method was grounded in years of study, experimentation, and detailed observations of the behavior of concretes made with aggregates with varying characteristics and origins. Through a thorough evaluation of widely used mix proportioning methods (such as Fuller [22], Shilstone [23], Viterbo O’Reilly [24], and ACI [25]), O’Reilly identified a shared limitation: these methods prioritize aggregate particle size while largely neglecting particle shape and environmental effects. This oversight calls into question their applicability, especially in cases involving aggregates with a high proportion of poorly sized or irregular particle shapes. Consequently, the traditional “ideal granulometric curve” proves inadequate, demanding a more comprehensive aggregate characterization. The limitations of conventional mix design methods are effectively addressed by the O’Reilly method, which tailors the concrete mix to the actual properties of the constituent materials. This approach enables the production of “custom-made” concrete with aggregate gradation for consistency, strength, and compaction. According to the author, this gradation saves over 10% on cement compared to traditional methods. Additionally, the Viterbo O’Reilly method significantly reduces the percentage of voids within the concrete, thereby enhancing durability and long-term material performance.

The Viterbo O’Reilly mix design is notable for its innovative approach to concrete mix with aggregate gradation. Unlike other methods, this one is based on a more detailed analysis of the materials’ properties and their behavior in the mix, which allows for obtaining concrete with specific and improved characteristics. One of the most important novelties of the Viterbo O’Reilly mix design is the ability to consider not only strength but also other factors, such as durability and workability of concrete. In addition, this method uses mathematical and statistical tools to adjust the proportions of the mixed components, resulting in a more accurate and efficient process. By leveraging a comprehensive and data-driven approach, it enhances concrete mixes more effectively than traditional methods.

This research is about the aggregate gradation enhancement of Portland cement concrete obtained with the Viterbo O’Reilly gradation method, in which compression, granulometry, specific gravity, and slump tests were used to understand the performance of the fabricated samples. Scanning electron microscopy (SEM) was used to examine the microstructure of certain selected samples. It is important to note that Viterbo’s method does not require a defined granulometry, making it suitable for determining the aggregate combination that minimizes voids while achieving the desired compressive strength. The primary innovation of this manuscript lies in the novel application of the Viterbo O’Reilly mix design method, which optimizes concrete mixtures through a detailed analysis of material properties and behavior, addressing a gap in the current research.

## 2. Materials and Methods

**Figure 1:**
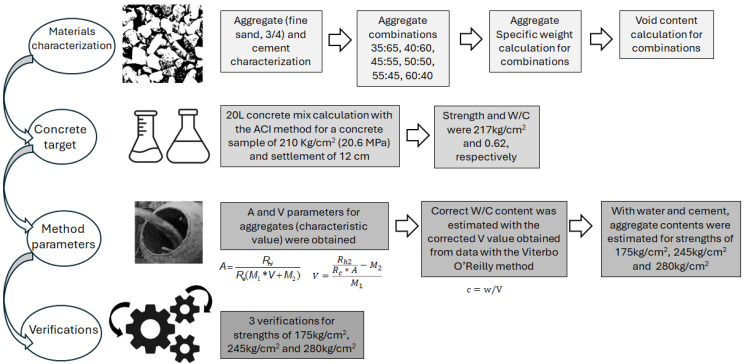
Summary of the Viterbo O’Reilly method as used in this research.

This research presents results of a University–Industry collaboration, a local strategy for implementing real solutions to large problems, with experiments and planning carried out in two locations, namely, the Universidad de Antioquia and the industry Conasfaltos, Medellín, Colombia. The main goal is to produce aggregate gradation enhancement that can lead to sustainable projects, from both environmental and economical points of view. The fabrication of samples and performance tests were conducted at the company Conasfaltos, while the materials characterization was carried out at the University. A civil engineering student moved permanently between the two places for discussion and tests. Periodically, the associated group for the project met to discuss the results and further strategies to implement the results at the company, on a large scale, using the company’s facilities and experience in large materials preparation. The mix design procedure utilized in this research was conducted using the Viterbo O’Reilly method for concrete aggregate gradation, which is summarized in Figure 1 and explained in detail below.

### 2.1. Materials

The aggregates selected for the mix were sand and 3/4” Piedra Verde gravel, both supplied by Conasfaltos S.A., Medellín, Colombia. The mix was designed to achieve concrete with a target compressive strength of 210 kg/cm^2^ after 28 days and a slump of 12 cm, values commonly required for structural concrete by the company. Therefore, the samples were based on experience in the field in the Colombia region, which enables them to be used in applications developed by Conasfaltos. Details of the mix design with materials and the experiments are presented in Table 1 and Table 2.

For the specified mix design, Type I Portland cement was used for both general use (G.U.) and structural use (S.U.) cement. The G.U. cement was obtained from one company, while the S.U. cement was sourced from another company, Company B, both located in Medellín, Colombia. A total of 350 kg of cement per cubic meter of concrete was used in the mix.

The main difference between general use (G.U.) or general purpose cement and structural use (G.U.) or high-strength early-age cement lies in the strength and the setting times with which they are produced. Structural cement offers accelerated strength development, which is perfect for projects requiring high strength quickly. General purpose cement, on the other hand, is more versatile and suitable for a variety of applications where very high strength is not required in a short period of time. General purpose cement (G.U.) is standardized by ASTM C1157 [28], while structural cement (S.C.) (type III Portland cement) is standardized by ASTM C150 [29].

### 2.2. Used Mix Design Method

The methodological design used in this research was proposed by Viterbo O’Reilly (1993) [24], which is used mostly in Cuba and in a few other countries. One of the main advantages of this method is the cement savings, which, indeed, compared to other methods (European, ACI), the O’Reilly method can reduce cement consumption by up to 15% concrete per cubic meter or more [24]. These savings in cement, of course, represent a very important economic and environmental impact for the planet and for the construction sector. The method, summarized in Figure 1, is explained in four steps: materials characterization, concrete target, method parameters, and verification process.

In the first step, materials characterization, as shown in Figure 1, the aggregates were studied. The aggregates (sand and 3/4” gravel) were first characterized based on the fineness modulus, granulometry, specific weight, and density. Both cement types, general use and structural, were analyzed. Then, the determination of weights was performed as follows: the volumetric and unit weights of the sand and 3/4” gravel were measured after drying them in a furnace. This allowed for the identification of the optimal gravel-to-sand ratio, with combinations of 35:65, 40:60, 45:55, 50:50, 55:45, and 60:40 tested. Then, the void contents and specific weight were calculated for each of these sand–gravel combinations.

In the second step, concrete target, as shown in Figure 1, the concrete mix preparation was made by using the ACI method. A 20 L concrete paste was mixed to achieve a 12 cm slump and a compression strength of 210 kg/cm^2^ (20.6 MPa). The water content necessary for this slump was determined, establishing the optimal water-to-cement ratio (W/C). Eighteen (18) cylindrical samples were fabricated using the optimized W/C ratio of 0.62, tested after curing for 28 days, and eighteen (18) mortar cubes were produced for each type of cement (general and structural use) and tested after curing for 28 days to evaluate the strength of cement. For the concrete mixes, compression tests were summarized with mean and standard deviation for each sample composition, based on three (3) tests.

In the third step, the Viterbo O’Reilly method parameters were calculated based on the data obtained from the concrete and mortar tests. The parameters, A and V, for the aggregates were found with Equations (1) and (2).

(1) $$A=\frac{R_{h1}}{R_{e}(M_{1}*V+M_{2})}$$

where A is the shape factor of aggregates, R_h1_ in the compressive strength mean value of the cylindrical samples; R_e_ is the strength of the cement after 28 days; V depends on W/C; and M_1_ and M_2_ are parameters related to the compressive strength and are calculated from the tables provided in the Viterbo O’Reilly method [24] (12 cm slump), where M1 = 3.9011 and M2 = 0.3780.

(2) $$V=\frac{\frac{R_{h2}}{R_{c}*A}-M_{2}}{M_{1}}$$

where R_h2_ is the compressive strength targeted; V is dependent on the water/cement ratio; and R_c_ is the compressive strength of the cement.

Then, the amount of cement was calculated for the strength and slump needed with all this information, as shown in Figure 1. And, with the amount of cement and water required, the gravel and sand amounts were calculated for 1 m^3^, with target strengths for both cements of 280 kg/cm^2^ (27.5 MPa), 245 kg/cm^2^ (24 MPa), and 175 kg/cm^2^ (17.2 MPa). The units of strength are presented in kg/cm^2^ because many companies work with them in many counties, but they are also presented in MPa for the international system of units. This makes this study inclusive for the two sectors, and it also allows for easy comparisons elsewhere.

The method concluded with three full verifications, ensuring the results were thoroughly validated through compression tests.

### 2.3. Materials Characterization

The morphological analysis of the concrete and aggregate samples was conducted with scanning electron microscopy equipped with energy-dispersive X-ray spectroscopy (SEM-EDS), manufactured by JEOL Ltd, Tokyo, Japan. All samples were sputtered with gold before the observation in a high vacuum. All tests were conducted at the University of Antioquia.

## 3. Results

### 3.1. Granulometric Analysis

**Figure 2:**
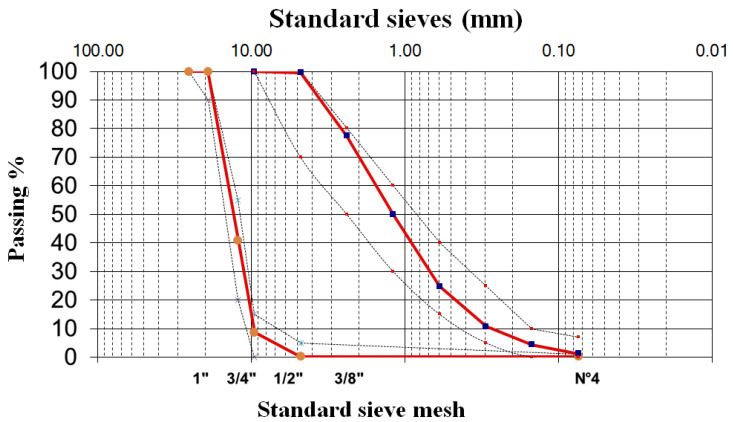
Granulometry data obtained for fine sand and 3/4” aggregate.

The granulometric test was carried out to determine the size distribution of the aggregate particles, both fine and coarse. The granulometric composition (or particle size distribution) of aggregates is crucial to the overall properties of concrete. It significantly influences key processes like mixing, transporting, placing, and compacting the concrete mix. The granulometry tests on the fine aggregate from the company Conasfaltos showed that the results were satisfactory because the fine aggregate complies with the granulometry in the sieves (8-16-30-50), which is shown in Figure 1. The results of granulometric analysis on the coarse aggregates of fraction 25-5 mm, carried out according to the standard, showed that the coarse aggregate from Conasfaltos is in compliance, in the ranges established under ASTM C702/C702M-18 [30], a standard practice for reducing aggregate samples to test size, and under ASTM C136/C136M-19 [31], a standard test method for mesh analysis for fine and coarse aggregate, as shown in Figure 2.

### 3.2. Specific Weight and Water Absorption

**Figure 3:**
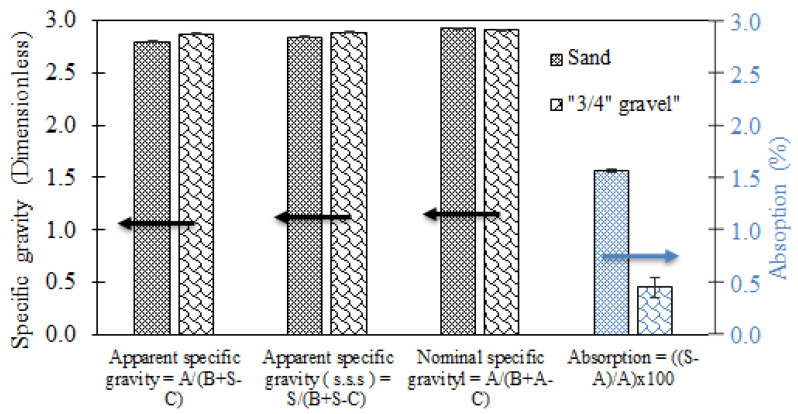
Weight and water absorption of sand and 3/4” gravel aggregates. A: Weight of the dried sample in air (g). B: Weight of the graduated pycnometer filled with water (g). C: Total weight of the graduated pycnometer with the sample and filled with water (g). S: Weight of the saturated sample (g), with a dry surface.

The specific weights and water absorption of the aggregates were obtained by weighing the sand in a dry state and saturated with water. In total, 1000 g of slag was obtained by quartering and then dried at a temperature of 110 °C in an oven to a constant weight. The aggregates were then immersed in water for 24 h, and once the time was up, they were poured onto a flat-bottomed tray to be dried in the open air. Next, the results were checked by filling a frustoconical mold, tamping it with the compaction rod, and giving it 25 blows. When removing the cone, the sample was evaluated; if it retained its shape, it was because it contained surface moisture. The process was repeated at frequent intervals, until (upon removing the truncated conical container) the sand cone collapsed. The experimental procedure was developed as follows: 500 g of material was introduced into a volumetric flask, and water was added until it reached the level. To remove the air bubbles, the volumetric flask was placed on a flat surface, tilted at about 30°, and rolled quickly over, holding it by the mouth until all the bubbles were expelled. This procedure was carried out for approximately half an hour. Then, water was added to the level, and the total weight was determined with an error of 0.01 g. The sand was extracted from the volumetric flask and dried to a constant weight at a temperature of 110 °C, allowed to cool, and weighed with an error of less than 0.01 g. Figure 3 shows the results obtained for the specific gravity and absorption. On the vertical left axis, this graph summarizes the specific gravity (in black, whose amounts are dimensionless, as shown in the formula on the horizontal axis), while it shows the absorption of the aggregates (%) on the vertical right axis (in blue).

The specific gravity in all estimated cases is quite similar, and it is near 3.0 for both samples, whereas the absorption is 1.56 and 0.46 for the fine sand and the 3/4” aggregate, respectively. In this image, A = air weight of the dried sample, in grams; B = weight of the volumetric pycnometer filled with water, in water; C = total weight of the volumetric pycnometer with the sample and filled with water, in grams; and S = weight of the saturated sample, surface dry, in grams.

### 3.3. Slump

The Abrams cone test was to determine the slump of the mixture, which was carried out by pouring the previously prepared mixture into a cone with standardized measurements and tamped with a steel rod with a pointed end through 25 penetrations. Then, the content was poured onto a flat surface and measured with a graduated ruler on the average sides and on the highest side, averaging the measurements. The results of the slumps by dosage are given in centimeters or millimeters, which are shown on the vertical axis in Figure 3 and discussed later in the results. Since the slump test was a predetermined variable, this study does not include its variability. This selection was better explained in the now improved procedure, as described below, which ultimately influenced the W/C ratios used in this study. For future research, we recommend including a study of comparisons of slump tests for sand and 3/4” gravel proportions, which certainly can lead to improvements in the workability and consistency of freshly mixed concrete, ensuring infrastructure applications.

### 3.4. Dosage of Mixtures

After determining the granulometry, specific weight and unit weight tests were carried out on the natural aggregates (fine and coarse) from an asphalt quarry. Several studies were conducted on Portland cement under compression to identify the quality of these components and thus to achieve the expected results with an optimal layout.

To determine the results of the design of the Hydraulic Concrete Dosages with a compressive strength of σ = 210 kg/cm^2^ (20.6 MPa), aggregates from the Conasfaltos quarry were used. The O’Reilly method was used, which determined the correct proportions of the aggregates based on the specific weights (both compacted and non-compacted) and water absorption.

To prepare the mixture according to the O’Reilly method, the optimal ratio of fine and coarse aggregates was determined by testing the mixtures of the aggregates with the proportions by weight of sand and gravel, as shown in Table 1. The compacted unit weight of the dry mixtures of sand and gravel was determined in the proportions suggested by the method. In order to determine the optimum ratio of the aggregate mixture, the aggregates were mixed in the proportions already indicated, and the compacted unit weight was determined for each mixture.

According to the O’Reilly method, after determining the compacted unit weight of the aggregate mixture (PUCm) by a standardized method, the ASTM C-29-78 standard [32] of each of the mixtures is expressed in Table 1.

**Figure 4:**
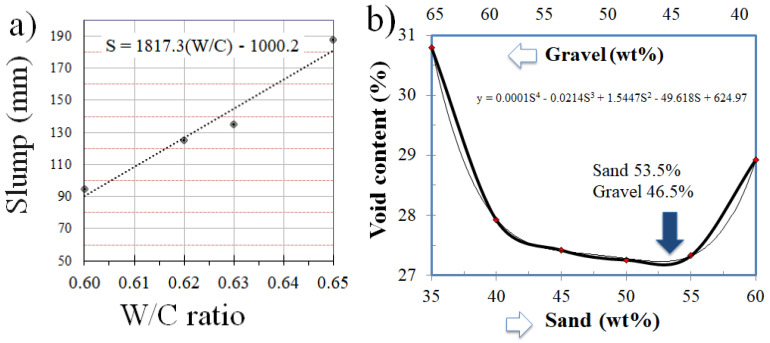
(**a**) Percentage of air void as a function of sand/gravel; (**b**) slump vs. W/C ratio.

The necessary amount of water was determined to prepare a concrete mixture with the required workability. With a slump of 9 cm measured in the Abrams cone, the workability test was repeated, until, by approximation, the total amount of water required to achieve the necessary slump was determined from the linear relationship shown in Figure 4a. Six specimens were made, and the compressive strengths of the specimens were determined after 28 days. These data were used to determine the coefficient “A”, which represents the characteristic of the coarse aggregate, as proposed by the O’Reilly method (see Equations (1) and (2)), which would help determine the appropriate percentages of gravel and fine aggregates, as shown in Figure 4b. The amount of cement to be used was determined using the equation proposed by the O’Reilly method. Finally, once the amount of cement and water was known, the amount of sand and gravel was determined according to the proportion of the optimal mixture, which is shown in Figure 4b, starting from the materials needed for 1 m^3^ of concrete with 2 or 3% trapped air.

### 3.5. Sample Manufacturing

The dosages used in this research were based on the O’Reilly method and took into account the humidity corrections made to the aggregates. Each combination was used in the preparation of 15 cm × 30 cm specimens and in a prefabricated element. Table 2 shows the dosage used to prepare these experimental mixtures for each type of cement, while Table 3 shows the optimal dosage for general and structural cement, which were replicated for both combinations.

To evaluate the properties of the concrete in the fresh state of both mixtures, rheological slump tests were carried out by the Abrams cone and by measuring the temperature at the time of concreting the prefabricated element.

For the evaluation of the specimens, 15 cm × 30 cm cylindrical molds were used, to which the concrete was added manually in three equal layers. Each mold was compacted by performing 25 rod strokes per layer to avoid voids and segregation of the concrete, as established by the standard. To prepare the test specimens, the molds were removed after 24 h for use with the next concrete combination.

To cure the specimens, the unmolded specimens were immersed after 24 h. The samples that were tested 24 h after being demolded remained in the curing tank for 2 h; then, they were dried superficially before carrying out the breaking process. The curing time was extended until the concrete reached ages of 3, 7, and 28 days for the rest of the specimens to be tested.

The mechanical behavior of the manufactured concrete was evaluated through compression tests, carried out in the laboratory of Conasfaltos, under the standards ASTM C39-20/C39M-20 [33], using a standard test method for determining the compressive strength of cylindrical concrete specimens, and under ASTM C78-18/C78M-18 [34], using a standard test method for determining the strength of concrete.

For the compression tests, three test cylinders of 15 cm × 30 cm were taken for each batch and for each mixture to be tested after 24 h and 3, 7, and 28 days, resulting in a total of 24 specimens, 12 made with general use cement and 12 made with structural use cement.

### 3.6. Compressive Strength

A comparison was made between the compressive strength results of concrete made with Portland cement for general use and Portland cement for structural use, with the objective of analyzing the properties and behavior of the material and its components against external stress to different curing ages.

**Figure 5:**
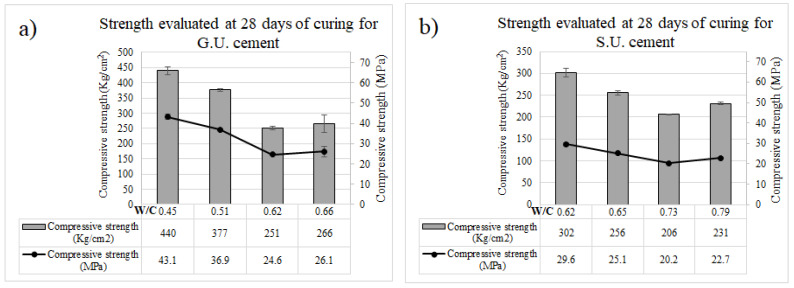
Samples made with different W/C ratios for (**a**) G.U. and (**b**) S.U. cements, all evaluated after 28 days of curing.

The strength results of the concrete for the first 24 h guarantee its use for the construction of structural elements that can be removed from a mold at an early age. The difference between the resistance of concretes made with general use Portland cement was, on average, 224.50 kg/m^2^ (22.0 MPa) for different W/C ratios, which demonstrates that concretes made with general use Portland cement exceed the resistance at early ages in comparison with those made with cement for structural use, which was 215.75 kg/cm^2^ (21.2 MPa), but even so, the superiority observed is minimal. Figure 4 shows the results of the resistance tests at 28 days for different W/C ratios. The values of the statistical analysis are shown in Figure 5. The average of both exceeds 205 kg/cm^2^ (20.1 MPa), which is the target resistance of the dosage designs. As W/C increases, in general, the compressive strength decreases, which is consistent with the increase in water and the corresponding defects, like voids.

The standard deviations were relatively small for all the samples, but, in general, they reduced with experience in the fabrication of samples since the samples leveled as 1 (G.U.1 and S.U.1) were made and tested first. As the repetition of sampling (verification samples) progressed, the error visualized via standard deviations was reduced, probably due to a better processing process (mixing and testing). Besides this small difference, the error bars were small, in all cases, less than 10% of the maximum strength.

**Figure 6:**
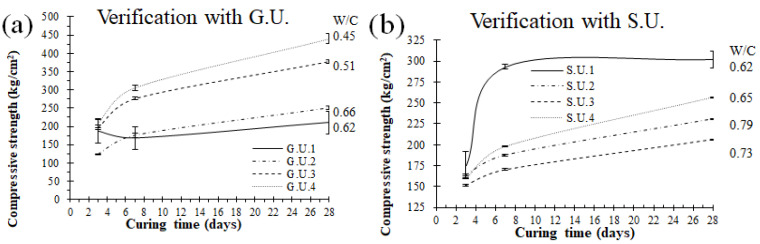
Samples and verifications made with different curing times for (**a**) G.U. and (**b**) S.U. cements.

This method started with cement and water amounts of 350 and 217 kg, respectively, for both the G.U. and S.U. cements estimated with the ACI method, with a W/C of 0.62 and a strength target of 210 kg/cm^2^ (20.6 MPa) for both types of cement. For the G.U. cement, the G.U.1 test showed 374 kg for 210 kg/cm^2^ (20.6 MPa). This means that for this specific cement type, the amount of cement increased by 7% with the method followed in this research. The verification tests (G.U.2, G.U.3, and G.U.4) gave cement amounts of 329 kg, 425 kg, and 482 kg for 175 kg/cm^2^ (17.2 MPa), 245 kg/cm^2^ (24 MPa), and 280 kg/cm^2^ (27.5 MPa), respectively. For the S.U. cement, the S.U.1 test showed 275 kg for 210 kg/cm^2^ (20.6 MPa). This means that for this specific cement type, the amount of cement decreased by 21.4% with the method used in this research. The verification tests (S.U.2, S.U.3, and S.U.4) gave cement amounts of 275 kg, 297 kg, and 334 kg for 175 kg/cm^2^ (17.2 MPa), 245 kg/cm^2^ (24 MPa), and 280 kg/cm^2^ (27.5 MPa), respectively. The results summarized in Table 4 and Figure 6 show that the target compressive strength (σ) was exceeded in most cases.

### 3.7. Scanning Electron Microscopy Characterization

**Figure 7:**
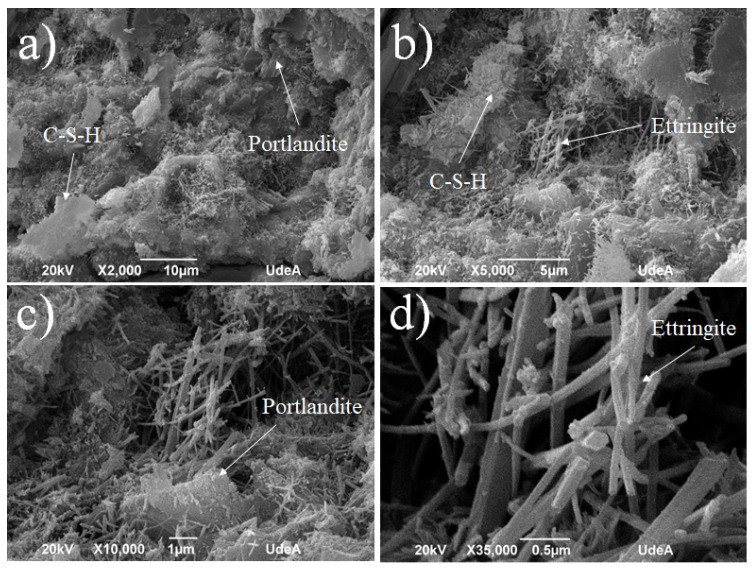
SEM for the G.U.1 cement sample at different zones and magnifications: (**a**) ×2000, (**b**) ×5000, (**c**) ×10,000, and (**d**) ×35,000.

The cement pastes for the general purpose design (G.U.) subjected to normal conditions at the age of 28 days show an extensive growth of C-S-H (calcium silicate hydrate) (Figure 7a,b) on which a large amount of ettringite crystals is observed (Figure 7c,d). Ettringite, a hydrous calcium aluminate sulfate mineral (Ca_6_A_l2_(SO_4_)_3_(OH)_12_⋅26H_2_O), is a product of Portland cement hydration. It contributes positively to the initial hardening of concrete, which can explain the high values of G.U. cement when compared with S.U. cement, as shown in Figure 6. However, if it forms later in the concrete’s life, it can cause destructive expansion and cracking. Likewise, the formation of large portlandite crystals is observed in the less dense areas, such as those shown in Figure 7d, which are found on the surface of an occluded air pore. A similar microstructure was observed before in concrete research [35,36,37].

**Figure 8:**
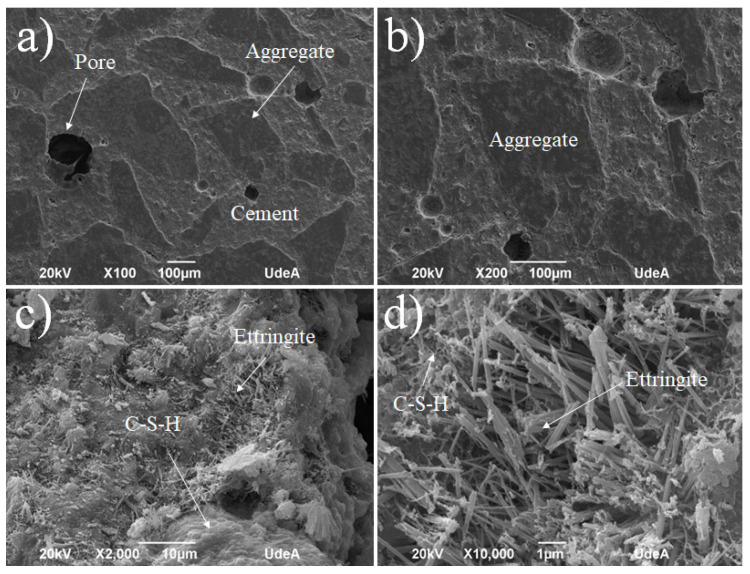
SEM for the S.U.1 cement sample at different zones and magnifications: (**a**) ×100, (**b**) ×200, (**c**) ×2000, and (**d**) ×10,000.

At the age of 28 days, the cement paste for structural use (S.U.1 sample) design shows a microstructure characterized by the formation of C-S-H with a compact appearance in some areas, although with pores in others, as can be seen in Figure 8a,b. Bar-shaped ettringite crystals are also observed (Figure 8c), along with some crystals with a prismatic appearance (Figure 8c) whose identification requires the use of other characterization techniques. Likewise, the formation of numerous portlandite crystals is observed in the form of massive groupings, as shown in Figure 8d. Besides the ettringite (Ca_6_A_l2_(SO_4_)_3_(OH)_12_⋅26H_2_O) formation, the pores appearing in Figure 8a,b for the S.U. cement can explain the lower values when compared to the G.U. cement, as shown in Figure 6. These pores increased with the W/C ratio, which means that they increased with the amount of water, which generated bubbles that could be trapped on the solid.

**Figure 9:**
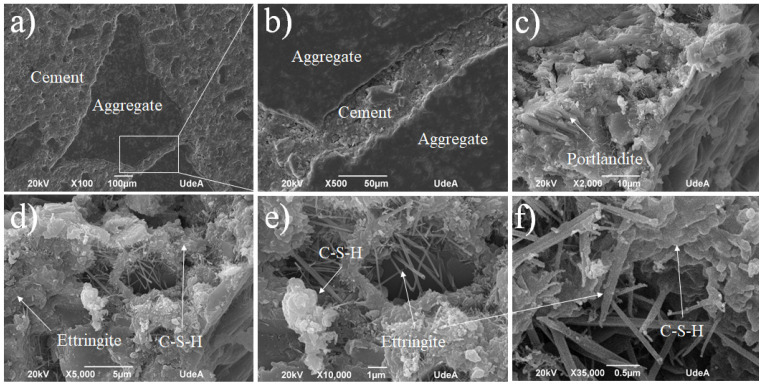
SEM for the G.U.2 cement sample at different zones and magnifications: (**a**) ×100, (**b**) ×500, (**c**) ×2000, (**d**) ×5000, (**e**) ×10,000, and (**f**) ×35,000.

At the age of 28 days, the verification sample G.U.2, corresponding to a general purpose cement, shows a quite compact structure without major pores, with the formation of C-S-H, covering the original cement grains and a large number of prismatic crystals, as well as ettringite crystals distributed throughout the fracture zone. This structure was observed through SEM, as shown in Figure 9a–c and, in detail, in Figure 9d. Large portlandite crystals were also notably present in the less dense areas of the paste (Figure 9e,f). A similar microstructure was observed before in concrete research [35,36,37]. These images confirm the results for the G.U.1 cement, as shown in Figure 6, without pores, which can explain the good compression test results.

**Figure 10:**
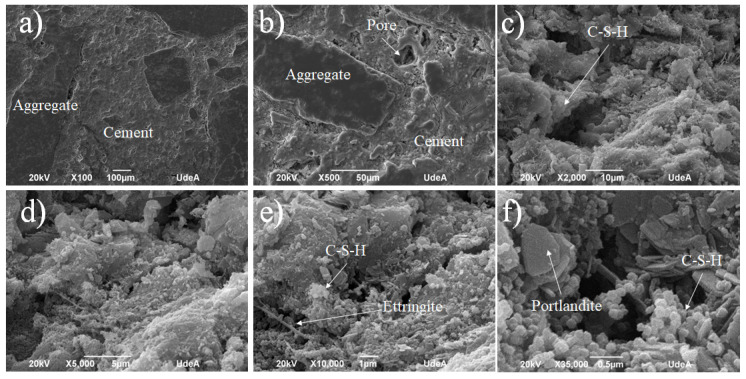
SEM for the S.U.2 cement sample at different zones and magnifications: (**a**) ×100, (**b**) ×500, (**c**) ×2000, (**d**) ×5000, (**e**) ×10,000, and (**f**) ×35,000.

Meanwhile, the verification sample S.U.2 cement paste with cement for structural use, subjected to normal conditions at the age of 28 days, shows the formation of numerous crystals in the form of clouds and intersecting plates forming an angle of 60° with each other, as shown in Figure 10a–d. This form of crystallization allows us to conclude that it may be an AFm-type phase formed as a consequence of the reaction between anhydrous aluminates and ettringite due to a decrease in the concentration of sulfates in solution. However, additional tests are required to confirm this aspect. On the other hand, large portlandite crystals are observed in the less dense areas of the sample (Figure 10f). These images confirm the results of the S.U.1 cement, as shown in Figure 8, with pores, which can explain the lower compression test results when compared to the G.U. samples.

**Figure 11:**
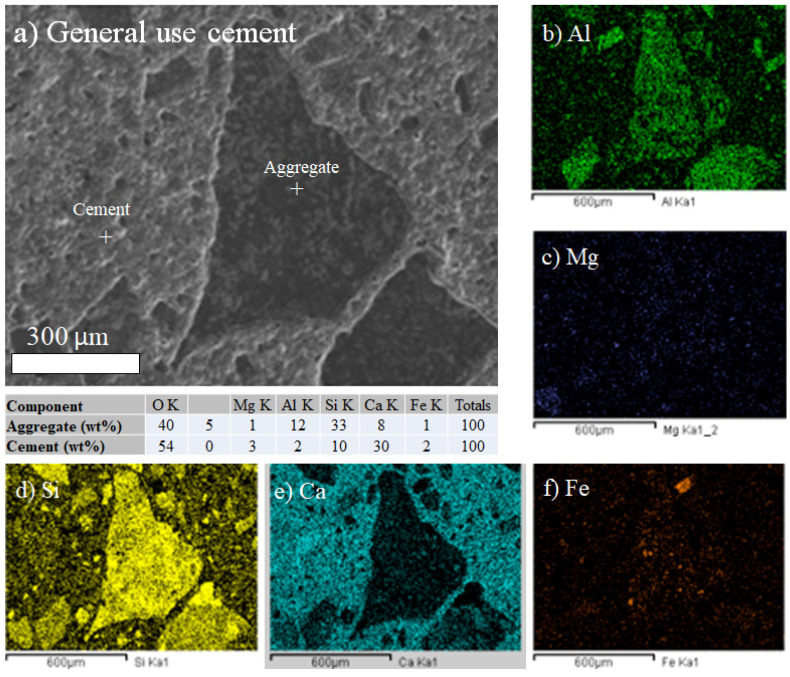
SEM-EDS composition maps for general use cement (G.U.): (**a**) SEM image, (**b**) Al map, (**c**) Mg map, (**d**) Si map, (**e**) Ca map, and (**f**) Fe map.

For the design with the general use cement, the distribution mapping of chemical elements on the good control sample surface was prepared. The aggregates mostly show aluminum and silicon, while calcium, mostly with silicon and aluminum, dominates in the cement matrix (Figure 11), corresponding to the calcium silicates and aluminates from Portland cement.

**Figure 12:**
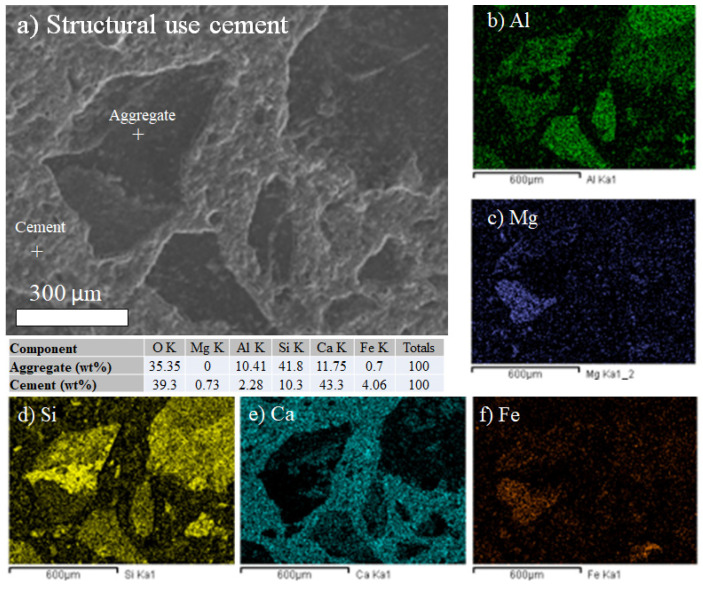
SEM-EDS composition maps for structural use Portland cement (S.U.): (**a**) SEM image, (**b**) Al map, (**c**) Mg map, (**d**) Si map, (**e**) Ca map, and (**f**) Fe map.

For the design with the structural use cement, the distribution mapping of chemical elements on the surface of one of the samples was prepared, which is evidenced by increasing damage. Silica and calcium are observed at the grain boundary; the remainder of the sample is occupied by magnesium. A homogeneous distribution of the elements is presented (Figure 12). Similarly, as shown in Figure 11, the maps confirm the presence of calcium silicates and aluminates in the cement matrix.

It is known that the shape of aggregates plays a key role in determining how efficiently particles fit together, which directly impacts the performance of the component materials in concrete. Shape types like angular, rounded, laminar, or elongated can significantly modify the packing density and the required quantity of cement paste. Angular aggregates, for instance, tend to interlock better, enhancing shear strength, whereas rounded aggregates are more likely to roll or slide, potentially reducing internal friction. Figure 11 and Figure 12 show, in detail, the shapes of the aggregates for the G.U. and S.U. cements, respectively, and although the SEM images represent only small areas (but with amazing details), they are useful in showing how the shapes can be related to the mechanical and gradation results for both cements. Both aggregate gradations presented in Figure 11 and Figure 12 show angular shapes, which can explain why the results, in general, were higher in compression strength than expected, as shown in Table 4. The angular aggregates in both images show better entanglement. Certainly, the aggregate shapes require a deeper study via optimization using not only electron microscopy but also tomography, which is proposed as future research.

The SEM-EDS analysis revealed the presence of Si, Al, Ca, Mg, Fe, and O in both types of cement. In general, these elements are all found in typical Portland cement; thus, because of their amounts, their presence does not necessarily indicate any adverse effects on hydration properties or long-term durability.

Common compounds include Tricalcium Silicate (3CaO·SiO_2_ or C3S), Dicalcium Silicate (2CaO·SiO_2_ or C2S), Tricalcium Aluminate (3CaO·Al_2_O_3_ or C3A), Tetracalcium Aluminoferrite (4CaO·Al_2_O_3_·Fe_2_O_3_ or C4AF), and gypsum (CaSO4·2H_2_O). Magnesia (MgO) can be added as an expansive agent to control shrinkage and reduce cracking [38]. When magnesium oxide (MgO) is added to concrete, it undergoes a hydration reaction with water to produce magnesium hydroxide (Mg(OH)_2_). This reaction causes a slight expansion in concrete, which can help offset the natural shrinkage that typically happens during the curing process. This is particularly beneficial in large structures, where shrinkage cracks are more likely to develop [39].

## 4. Discussion

This project showed a potential successful solution to reduce costs in research and the development of projects involving large-scale industries that could have an impact on the environment. The problem of aggregate gradation enhancement, as discussed in the Introduction, has an impact not only on material properties but also on the environment, as concrete cement or concrete asphalt requires aggregate gradation to be enhanced to have a lower CO_2_ footprint and costs [40,41]. The topic of sustainable construction is a common interest for new students [42]. There is also an increasing need to reduce cement and pollution, which can be seen in the construction industry’s proposal to use new materials [43] or recycled aggregate in concrete [44]. In addition, an important and high-impact strategy is reducing the amount of cement [45] and water [3] in concrete. Therefore, the project described in this research demonstrates a model to follow, particularly when reducing costs is important, which is a very common problem in many nations where companies have cost and material limitations. The resources for this project, encompassing university and company facilities along with the student′s basic salary, were already secured. This investment proved crucial, culminating in a significant and sustainable solution with the potential for large-scale application within the company. This type of solution, involving university and industry collaboration, has been reported successfully before, with some variations, but, in general, it provides important results for the solution to large-scale problems [46,47].

As mentioned above, the Viterbo O’Reilly gradation method followed in this research has been poorly researched [24], showing important results, particularly when S.U. cement was used, which produced cement savings of 21.4%. Previous works on concrete using aggregates processed with the Shilstone gradation presented very different results, mostly focused on reducing voids and thus increasing compressive strength. This is the case of a study on Wisconsin pavements [23], which reported water savings of up to 15%, a 20 to 30% reduction in void content, a 10 to 20% increase in compressive strength, and a slight increase in cement content. Thus, rather than the material enhancement and water reduction, not many savings in costs were obtained. Another study focused on the durability of concrete, using Shilstone gradation as well [48], but with quite low W/C (between 0.30 and 0.40) and slump (between 3 and 8 cm). The results showed some improvements in the targeted variables, but no information about cement or water savings was provided, which shows the regular focus on performance properties in these studies. However, one study from Nigeria used Shilstone gradation in concrete and focused on sustainability [49]. The study considered similar target strengths as those investigated in this research (300 and 350 kg/m^3^) and found a slight decrease in compression strengths but potential savings in cement of up to 11%. On the other hand, a Fuller study investigated concrete durability [50], finding important improvements (particularly when a modified Fuller method was used) not only in compressive strength and durability but also in using additives. However, cement and water savings were not mentioned in the study. Another study using Fuller gradation focused on sulphoaluminate cement concrete [51], developing formulations with improved water permeability, compressive strength, and resistance to sulfate attack. Nevertheless, cement and water savings were not mentioned in the study. Finally, some other studies focused on cement reduction, but they did not follow any standard method. Some of them reported good results when using aggregates to reduce cement in concrete (reporting cement savings up to 30% and cost savings ranging from around USD 1.30 to USD 10.50 per cubic meter of concrete) [52]. However, as explained in this study, an increase in aggregates limits the workability and, thus, the applicability of the mix.

The compressive strength of the developed formulations is highly suitable for various structural applications, such as housing, side roads, barriers, and more. This aligns with the standards outlined in Standard Specification for Unit Masonry [53], which serves as a guideline for classifying, specifying properties, and determining the applications of cement–lime mortars. According to the standard, the minimum average compressive strength values for different cement types are as follows: M (17.2 MPa), S (12.4 MPa), N (5.2 MPa), and O (2.4 MPa). These types are used in specific building applications: load-bearing, parapet walls, non-load-bearing walls, tuckpointing, foundation walls, retaining walls, pavements, manholes, sewers, walkways, and patios, among others. Given that the formulations in this research demonstrated a mean compressive strength of more than 100 MPa, they can be improved for use in all these applications.

It is important to note that the Viterbo O’Reilly design method focuses on enhancing compressive strength via aggregate gradation by analyzing void content curves across various aggregate gradations. The ideal aggregate blend is identified by achieving the lowest volume of air-filled voids. However, this method has certain limitations: it is not widely studied and thus, not well understood, and it does not account for key variables that directly or indirectly affect factors such as workability and pumpability.

The Viterbo O’Reilly method is an aggregate gradation process that improves the compressive strength and materials (water and cement content) of the aggregate mix, but it is not an optimization process itself, so it could be further improved if combined with modern optimization techniques with computational work.

On the other hand, the effect of aggregate size on concrete is a well-researched topic. It is known that a smaller aggregate size can increase the initial compressive strength, while larger aggregates can increase long-term strength and durability. Gradation enhancement can be important because it leads to a reduction in the amount of cement and voids while improving durability, strength, and workability, among other properties [54,55].

The method applied in this study revealed that, for the specific G.U. cement type, cement usage increased by 7%. In contrast, for the specific S.U. cement type, there was a notable reduction in cement content by 21.4%. While all aggregate combinations met the required standard strength, the S.U. cement type demonstrated particularly significant potential for cement savings. While target strengths were obtained in all cases, curing and early strengths were not investigated in this research. Considering that this information is important for multiple applications, including precast concrete, we recommend conducting future research. As a vital next step, upcoming research should include a life cycle analysis comparing traditional and optimized methods. This will be a significant outcome for assessing the method′s effectiveness. In general, the obtained compressive strength values were higher than expected (6 out of 8), so these results were important from a properties point of view. In addition, the method is rather influenced by the cement type, as shown in this research (cement contents decreased for the G.U. cement while they increased for the S.U. cement), suggesting chemistry may play an important role.

Taking into the account that the method shows, specifically for the S.U. cement type, that the amount of cement was decreased by 21.4%, that nearly 1 ton of CO_2_ can be released to the atmosphere as a byproduct of each ton of manufactured cement [4], and that about 4 billion metric tons of cement are made annually, about 0.86 billion metric tons of cement could be virtually saved. Thus, the main result of this research shows that the method used here can result in up to 21.4% savings in costs due to cement savings, which is very significant for any construction project.

The graphical representation of the gap-to-gravel ratio in Figure 4a is insightful, considering the well-known and meaningful effect of voids on the structural properties and durability of concrete [56], which is particularly important in civilian infrastructure, where construction may stand for a long time. From a materials perspective, a void decreases the strength of the material acting as a stress concentrator [57], which is even more critical in concrete, which is a fragile material where crack growth is fast and catastrophic due to its low fracture toughness [58]. Therefore, a goal of construction is to reduce voids to a minimum [59] (and thus, to increase density as well) to increase durability and strength. This reduction in voids impacts other important factors, such as decreasing infrastructure costs (by increasing building life and reducing concrete maintenance) and reducing environmental pollution (by reducing the cement used when construction life increases, thus lowering the CO_2_ footprint). There are some cases where voids in concrete are also wanted (with the corresponding limited strength and durability), such as in applications requiring a light weight [60], thermal insulation [61], and noise insulation [62].

The water-to-cement ratio (W/C) of 0.62 obtained using this method and tested for the formulations is relatively high, as a ratio between 0.40 and 0.60 is used for general use (G.U.) and structural use (S.U.) concretes [63]. However, for the slump value of 12 cm, and for the local cements tested, the value meets the initial requirements, as successfully tested by the company as well, but with potential limitations for high strength [64]. Further tests must explore other combinations of aggregates and materials, including additives [65], which could help to reduce W/C without compromising substantially properties like workability and fluidity [66].

The results shown in this investigation (compression strength, void content, cement and water decrease, and others) can be potentially improved by using additives [67,68] and admixtures [69], with pozzolanic activity [70] or functional properties, including nanoparticles [71], which can adapt the method to other products, such as ultra-high performance concrete (UHPC) [72], ultra-high-performance fiber-reinforced concrete (UHPFRC) [73], or high-temperature cements [74].

## 5. Conclusions

This research has shown the results of University–Industry projects, with potential to develop sustainable solutions on a large scale, particularly those carried out with experiments that can be validated on a large scale. Since the experiments were conducted in two places, Universidad de Antioquia and the industry Conasfaltos, the aggregate gradation can lead to really sustainable projects, as costs are also reduced. The main findings are summarized below.

- There is little data available on the Viterbo O’Reilly method, possibly because of the poor reports and lack of research on it. However, this research shows that it is a valid method, feasible for use with good results.
- The Viterbo O’Reilly method showed very different results for each of the cements. For the G.U. cement, the G.U.1 test showed 374 kg for 210 kg/cm^2^ (20.6 MPa). This means that for this specific cement type, the amount of cement increased by 7% with the method followed in this research, when compared with the 350 kg cement estimated by the ACI method. The verification tests (G.U.2, G.U.3, and G.U.4) gave acceptable strengths. For the S.U. cement, the S.U.1 test showed 275 kg for 210 kg/cm^2^ (20.6 MPa). This means that for this specific cement type, the amount of cement decreased by 21.4% with the method used in this research, when compared with the 350 kg cement estimated by the ACI method. The verification tests (S.U.2, S.U.3, and S.U.4) gave acceptable strengths.
- The compressive strength behavior of concretes manufactured for structural use is lower than that manufactured with cement for structural use, but even so, it falls within the permissible range for the target resistance of 210 kg/cm^2^.
- The tests were carried out with a reliability level of 95% for all the cases studied, and the standard deviation values confirm that there is no notable dispersion between the resistance results obtained for each tested specimen. With the application of the experimental method for dosing materials with the Viterbo O’Reilly method, it is possible to obtain concrete with the lowest use of the necessary aggregates.
- SEM images show very low porosity and, in general, a good aggregate–cement relation. The amount of C-S-H formed at the age of 28 days is similar in the two designs studied, indicating similar hydration levels in the two samples. Likewise, at this age, the pastes show microstructures characterized by extensive growth of C-S-H, whose main differences lie in the morphology, composition, and size of the different crystalline phases, which, in principle, do not contribute significantly to the development of resistance.

## Figures and Tables

**Table 1:** Sand and gravel formulations used in this research.

Sand (%)	65	60	55	50	45	40
Gravel (%)	35	40	45	50	55	60
Total (%)	100	100	100	100	100	100

**Table 2:** Material dosages used.

Sample Type	Sample Name	Cement (kg)	Water (kg)	W/C	Sand (kg)	Gravel 3/4” (kg)
General use Portland cement (G.U.)	G.U.1	350	217	0.62	955	896
	G.U.2	329	217	0.66	945	822
	G.U.3	425	217	0.51	897	780
	G.U.4	482	217	0.45	869	755
Structural use Portland cement (S.U.)	S.U.1	350	217	0.62	955	896
	S.U.2	275	217	0.79	972	845
	S.U.3	297	217	0.73	961	835
	S.U.4	334	217	0.65	943	819

**Table 3:** Mix design of the cements and their slump of 12 cm.

Parameter	General Use (G.U.) Cement	Structural Use (S.U.) Cement
Water (kg)	217	217
Cement (kg)	374	275
Aggregates (kg)	1720	1813
Gravel (kg)	800	843
Sand (kg)	920	970

**Table 4:** Summary of results and concrete designs.

Samples	W/C	Cement (kg)	Water (kg)	Sand (kg)	3/4” Gravel (kg)	As (cm)	σ (kg/cm^2^) Targeted	σ (kg/cm^2^) Obtained
G.U.1	0.62	350	217	955	896	12	210 (20.6 MPa)	211 (20.7 MPa)
G.U.2	0.66	329	217	945	822	10	175 (17.2 MPa)	251 (24.6 MPa)
G.U.3	0.51	425	217	897	780	10	245 (24 MPa)	377 (37.0 MPa)
G.U.4	0.45	482	217	869	755	70	280 (27.5 MPa)	440 (MPa)
S.U.1	0.62	350	217	955	896	12.5	210 (20.6 MPa)	315 (43.1 MPa)
S.U.2	0.79	275	217	972	845	10	175 (17.2 MPa)	231 (22.7 MPa)
S.U.3	0.73	297	217	961	835	14	245 (24 MPa)	206 (20.2 MPa)
S.U.4	0.65	334	217	943	819	17.5	280 (27.5 MPa)	256 (25.1 MPa)

## Data Availability

The data presented in this study are available on request from the corresponding author due to raw data contains private information from Company-University.
